# Supplementary material for: Active search strategies, clinicoimmunobiological determinants and training for implementation research confirm hidden endemic leprosy in inner São Paulo, Brazil
Source: PLoS Negl Trop Dis. 2021 Jun 14;15(6):e0009495. doi: 10.1371/journal.pntd.0009495 (PMC8224878; doi:10.1371/journal.pntd.0009495)
Supplement: Attachment — Submitted filename: Author Responses May 05, 2021.docx [file pntd.0009495.s001.docx]

May 05, 2021.

Paul J. Converse

Associate Editor

PLOS Neglected Tropical Diseases

Ana LTO Nascimento

Deputy Editor

PLOS Neglected Tropical Diseases

Sub: PNTD-D-21-00357

Dear Editors,

We would like to thank you for all comments about our manuscript: “Active search strategies, clinicoimmunobiological determinants and training for implementation research confirm hidden endemic leprosy in inner São Paulo, Brazil” because they appointed some important aspects to improve it.

The requested edits were considered and they are in a red in the manuscript.

**Remarks**

**Reviewer's Responses to Questions**

**Methods**

-Are the objectives of the study clearly articulated with a clear testable hypothesis stated?

-Is the study design appropriate to address the stated objectives?

-Is the population clearly described and appropriate for the hypothesis being tested?

-Is the sample size sufficient to ensure adequate power to address the hypothesis being tested?

-Were correct statistical analysis used to support conclusions?

-Are there concerns about ethical or regulatory requirements being met?

Reviewer #1: The objectives are clear and the study is well-designed. The sample size and statistical analyses are well-fitted to the study. There are no ethical concerns.

Author’s comments: Ok.

Reviewer #2: The objectives of the study are clear and well presented.

Author’s comments: Ok.

Reviewer #3: 1.Abstract/Summary

Line: 38 "All patients were multibacillary and presented hypochromatic macules with loss of sensation"

Author’s comments: Yes, they are.

**Results**

-Does the analysis presented match the analysis plan?

-Are the results clearly and completely presented?

-Are the figures (Tables, Images) of sufficient quality for clarity?

Reviewer #1:

The results are clearly presented. My only concern is that the paper is quite long and some of the results could be regarded as superfluous, including the photos of skin lesions (Figures 2 and 5), and the follow-up data in Table 7 (this is a paper about improved case-finding).

Author’s comments: Yes, we agree with you about the size of the paper, but we worked a lot with a considerable population sample and for a long time. About clinical photos, we thought they are important because we would like to highlight and to warn about the macular manifestations of the leprosy patients, because a few number of papers have talked over it, but it is the most frequent manifestation of leprosy nowadays. Every paper shows us always the same pattern of leprosy presentation considering mainly the scientific Ridley & Jopling classification, that does not consider this kind of manifestation, but it represents the clinical current reality of leprosy on the field. About the Table 7, we just put this table to show for the health managers the significant interference on these data when the health team decide to work (after 2015 on the table) properly with leprosy making active search actions focused on a broad view of the disease considering aspects that are not only cutaneous, but also neurological, and changing completely the tendency line of the municipality.

Table 5 shows that measuring anti-PGL-1 antibodies is of no value in establishing the diagnosis of leprosy, so Figure 3, which shows the same data, is not needed.

Author’s comments: We decided to erase the Figure 3, adding only the mean values of anti-PGL-I OD in both groups in the first paragraph before the Figure 3 on page 16.

“Considering anti-PGL-I antibody results, 40.6% were positive in LG (26/64) while 32.3% in NLG (134/415), an RR of 1.4 (95% CI 0.89-2.16, p=0.19); LG presented higher OD titer mean (0.248) than NLG mean (0.180) and p=0.03.”

In general, prevalence rates are no longer of much importance, while new case detection rates indicate more clearly what the epidemiological situation is. In this case, however, where the study is in a small town (pop ~ 40,000) actual case numbers should be given, rather than rates - this applies especially to Table 7, as the real catchment population is not known; people may have heard of better services being available and may have travelled from other surrounding towns.

Author’s comments: Thanks a lot for this comment. We agree with you about the prevalence rate, but we decided to consider both, and we included the actual number of cases by year changing also the name of Table 7.

The mapping information is not very well analysed and is perhaps something of a distraction from the main message of the paper.

Author’s comments: About the mapping we have many other points to consider, but the most important for the authors was to demonstrate visually that the disease is distributed throughout the town, and not only in the west region defined unfortunately by us as a leprosy cluster in the paper published before about the town (Bernardes F Filho, Paula NA, Leite MN, et al. Evidence of hidden leprosy in a supposedly low endemic area of Brazil. Mem Inst Oswaldo Cruz 2017; 112: 822-8. https://doi.org/10.1590/0074-02760170173 PMCID: PMC5719551).

Reviewer #2: Results are properly presented

Author’s comments: Ok.

Reviewer #3:

1. line 197 "Although the CHAs had theoretical training, no questions specific mentioning leprosy were 198 posed to respondents, who were instead asked to answer general questions about signs and/or 199 symptoms and to return the LSQ to CHAs." But the title of the questionnaire is the "leprosy suspicion questionnaire" so I am not quite sure what you are signalling here? Are you trying to avoid anxiety amongst the responders about the diagnosis of leprosy?

Author’s comments: Thank you for comments. Really our writing was not clear. We changed the text because we should consider that for this paper the CHAs have applied the questionnaire individually for most of the population avoiding bias about leprosy, and they did not have contact with the questionnaire physically, they just answered the questions made by CHAs. Because your questions we changed the text lines, changing mainly the verb “distributed” for “applied”.

2. Esthesiometry is not a familiar term to me so I looked it up. It appears to mean the technique of measurement of sensation.

I think you mean "loss of sensation" or “anaesthesia” might these be more universally understood terms?

Author’s comments: Thank you for your comments. I agree with you and the term may be unfamiliar, but correctly the term esthesiometry means the technique of measurement of sensitivity (esthesio).

3. So why is the questionnaire not picking up paucibacillary patients. Is it obvious why? Perhaps you might include at some point in the manuscript your thoughts about this.

Author’s comments: Thanks for your comment. Coincidently, we did not get any paucibacillary patients, but our goal was just to improve the chance of leprosy diagnosis independently the classification. Considering mainly that the neurological symptoms and skin signs may be present in pauci and multibacillary forms of leprosy and considering that we did not find any paucibacillary patient, we judged not appropriated to mention about it in the manuscript.

4. Would it be useful to indicate roughly how long the LSI took to complete? Ie the relative cost of including this within a future Public health screening approach?

Author’s comments: Thanks for your comment. Effectively, the CHAs spend a relative short time to complete the LSQ. About the relative cost to include LSQ within a future Public Health screening approach is low considering its effectiveness as demonstrated in this paper, but one important point to consider is a need to have a health team trained in leprosy to recognize the disease in its frequent and challenging macular forms.

**Conclusions**

-Are the conclusions supported by the data presented?

-Are the limitations of analysis clearly described?

-Do the authors discuss how these data can be helpful to advance our understanding of the topic under study?

-Is public health relevance addressed?

Reviewer #1: The main conclusions are that the Leprosy Suspicion Questionnaire is a valuable tool for active case-finding, as it brings into better focus the neurological aspects of leprosy, and that this helps in the training of health workers to be more aware of this component of the diagnosis of leprosy. This is an important message.

Author’s comments: Ok.

The authors mention two limitations of the study, but do not discuss any further implications for public health. The study was only able to evaluate 300 of the 1,054 LSQ+ respondents, for reasons that are not explained, but this is quite a serious flaw in the study. They also say that they were unable to reevaluate people who did not have leprosy but who were anti-PGL-1 positive. It seems to me that the LSQ is a very useful tool for case-finding, with high sensitivity, while testing for anti-PGL-1 antibodies is shown in this study to be an extremely poor diagnostic test for leprosy.

Author’s comments: Thanks for your comments. In fact, it was a pity that we were unable to evaluate all the respondents, but originally, we thought just to use these respondents for the practical training of the primary health teams. Also, about the anti-PGL-I positive individuals, we did not get to evaluated all of them because difficulties in relation to municipal health management.

Considering that leprosy is primarily a neural disease, neurological symptoms appear to be more evident and precocious in LSQ. On the other hand, the anti-PGL-I represents an immunological biomarker related to mainly with bacillary load which corroborating with our low number of patients with PCR positive results put this marker as a poor tool for the leprosy diagnosis.

It is surprising that the authors do not make any recommendations, or even suggest how they may plan to use the LSQ in future. For example, they have shown that five of the fourteen questions in the LSQ are more indicative of leprosy, so a reasonable approach may be to use a 'short' questionnaire, as part of an organized campaign in which all LSQ+ people are evaluated. This could be done in many different contexts, such as a skin camp, or a health education campaign, etc. Abandoning the use of anti-PGL-1 testing may free up resources to properly assess LSQ+ respondents.

Author’s comments: Thanks a lot for your comment and suggestions. We added this suggestion on the end of first paragraph of conclusion (page 25): “… The LSQ also functioned as a consolidated, simple, cost-effective facility for health education, renewing awareness of leprosy signs and symptoms in the collective consciousnesses of the population and health professionals, and it should be recommended in the routine of screening and surveillance actions, complete or at least with the five most significant issues for the diagnosis of leprosy.”

Reviewer #2: Conclusions are in line with the paper

Author’s comments: Ok.

Reviewer #3:

1. 384 leprosy presence working at reference, I don’t understand this sentence. Can you reword?

Author’s comments: Thanks for your comment. This sentence was reworded for: “Collection of follow-up data was possible because the presence of dermatologist experienced in leprosy working at reference…”

2.Do you foresee this LSQ being used operationally as a screening tool by your public health teams? I thought this was what you intended but your focus at the end of the manuscript is of the benefit in involving CHA in research. I would have thought there is also potential benefit for case detection able it one that will not identify all cases, and possibly not paucibacillary disease.

Author’s comments: Yes, we do. About the paucibacillary disease, please have a look on the comments above.

**Editorial and Data Presentation Modifications?**

Use this section for editorial suggestions as well as relatively minor modifications of existing data that would enhance clarity. If the only modifications needed are minor and/or editorial, you may wish to recommend “Minor Revision” or “Accept”.

Reviewer #1: As mentioned above, the authors seem keen to present every last piece of data that they collected, sometimes to the detriment of the overall message. I think the questionnaire they have developed is a very important advance for case-finding.

Author’s comments: Thanks for your comments. You are right, because the project was really long and large and many results were obtained, however, in addition to the questionnaire, other results were important, such as undoing the definition of a cluster given to a region of the town in our previous publication and demonstrating that the whole town has a similar risk of falling ill by the hidden endemic. In addition, the valuation of macular lesions for the diagnosis of leprosy cases, the results of the implementation of actions mainly related to the training of professionals, and the long clinical follow-up of patients by the same dermatologist.

Also Ref 14, which is said to be in press, is now available: Bernardes Filho F, Santana JM, de Almeida RCP, Voltan G, de Paula NA, Leite MN, et al. (2020) Leprosy in a prison population: A new active search strategy and a prospective clinical

analysis. PLoS Negl Trop Dis 14(12): e0008917. https://doi.org/10.1371/journal.pntd.0008917

Author’s comments: Ok. We updated this reference.

Reviewer #2: only more information and discussion on SSS

Author’s comments: Thanks for your comment, but we did not perform the SSS for microbiology study, just we have used skin slit smears just for RLEP-PCR, and considering the low percentage of positivity among our patients and the already quite long results to preset we did not consider additional information about it.

Reviewer #3: (No Response)

Author’s comments: Ok.

**Summary and General Comments**

Use this section to provide overall comments, discuss strengths/weaknesses of the study, novelty, significance, general execution and scholarship. You may also include additional comments for the author, including concerns about dual publication, research ethics, or publication ethics. If requesting major revision, please articulate the new experiments that are needed.

Reviewer #1: There is nothing specifically wrong here, but much of the material presented is irrelevant to the main message of the paper, which is the value of a questionnaire focusing on neurological symptoms in combination with training of health staff on this aspect of leprosy diagnosis. The title of the paper could be more eye-catching, such as "The Leprosy Symptom Questionnaire (LSQ) focuses on common neurological sypmtoms of leprosy and is a highly effective case-finding tool."

Author’s comments: Thank you for this comment. We understand your enthusiastic feeling about the LSQ but it does not have only symptoms, we considered also many signs related to leprosy. This paper we demonstrate the effectiveness of LSQ on the community, different of this rule in a prison population as published before (Bernardes-Filho 2020). Because of this, we cannot change the title like your suggestion.

Reviewer #2: Dear Authors,

I read your paper with interest having read the previous paper about this town and the use of the questionnaire in a prison population. I am impressed about the results the questionnaire gave.

It was a particularly good choice to use neurological symptoms, because there is no leprosy without nerve involvement.( Fite GL. 1943. Leprosy from histopathologic point of view. Arch Pathol Lab Med 35:611–644.)

The paper on the questionnaire contains at the end a second part on the spatial distribution. I can not find this in the abstract.

Author’s comments: Thanks for your comments. About the reference, we have added this in the manuscript, lines 315-316: “Diagnoses are closed only after SSS and/or biopsy; neurological symptoms are unfortunately not considered, although there is no leprosy without nerve damage, like Fite stated in 1943 [22].”

About the abstract, we really did not put about spatial distribution because of the space, but we changed same words and we have added a short information about it in the end of the findings part: “… The NC and anti-PGL-I spatial mappings demonstrated the disease spread across the town.”

I have a few questions and remarks:

79 deformities are muscle wasting contractures and absorption, I doubt injuries.

Author’s comments: Thanks, but I did not know about your doubt. We changed the sentence for: “This bacterium affects peripheral nerves and gives rise to deformities such as muscle wasting and wounded lesions over anaesthetized areas of the body [3].

87 even under dermatologists

Author’s comments: Thanks. We added it.

Training not trainings

Author’s comments: Ok, we changed it. Thanks.

146 I assume that SSS was used for diagnosis with AFB staining.

Author’s comments: Traditionally yes, however, we can use the SSS for more sensitivity – PCR.

157 I see AFB was done.

Author’s comments: No, AFB was not done in this study.

167 positive PGL1 does not mean infection, just contact. Thus, better use this instead of subclinical.

Author’s comments: Thanks a lot. We cut of this subclinical infection term changed for:

80 - “To produce maps of leprosy cases distribution in the town, we used QGIS 3.10.7-A Coruña (http://www.qgis.org).”

206 How where the positive responders selected for clinical evaluation?

Author’s comments: They were randomly selected.

209 you mention both groups the LG and NLG from the 300?

Author’s comments: Yes, both groups among 300 LSQ+ respondents evaluated clinically.

212 How were the non-responders selected for clinical evaluation?

Author’s comments: Also, they were randomly selected.

In table 4 I expected positive SSS for AFB. But there was none?

Author’s comments: We did not perform SSS for AFB.

259 How many had a positive AFB when, so few had a positive PCR? What I do not understand, did you not find a positive SSS? And you had 64 MB patients, did you classify only on the number of patches.? How many patches had no sign of nerve damage? In LLs and may be BL, I would expect only the early patches with detectable damage. You did it very carefully. In a borderline group you usually find patches with loss. But not all. How many did you test with no detectable nerve damage?

I liked the street view with Leprosy patients and PGL1 positive contacts.

The primary healthworkers seemed well trained!

Author’s comments: Thanks for your comments. We understand your doubts and we will try to explain about our goals in this study. Firstly, we did not do SSS for AFB. We performed RLEP-PCR with more sensitivity, but in this case seemed not so efficient. About our classification, we did not perform it based only on the number of patches, but always testing many of them. Additionally, most of them had infiltrated patches and also we evaluating all patients neurologically. On the table 4, we have found 72% of patients with altered nerve palpation and 86% of abnormal feet esthesiometry, corroborating of multibacillary classification of patients.

Thanks for the last comments about street view and training.

316 from here I understand again that diagnosis was confirmed after SSS. Like you mentioned in the introduction why can I nowhere find the result? My fault? For me 64 MB patients and no positive SSS is strange.

Author’s comments: We did only SSS for RLEP-PCR and unfortunately, we had only 8.9% of positivity.

345 Good to mention here that among the women 11% was positive and among the male 12.3% So the man/women may be in line with the country’s findings.

Author’s comments: Yes, thanks.

353 Here I find a mention of SSS. How would you classify your patients?. Ridley Jopling BT or old Brazilian Inditerminate? Could you check staining of your SSS AFB. Because M.leprae is only weakly acid fast. May be that count for your negatives. At your institite (in Ribeiroa Preto) it will be done properly but where were the SSS stained? In a TB lab? I do not doubt that 100% of the clinical diagnosed patients were leprosy patients. But in my experience not 80-100% of macular leprosy in Brazil are SSS negative.

Author’s comments: We did only SSS for RLEP-PCR and unfortunately, we had only 8.9% of positivity.

Reviewer #3: Thank-you for the opportunity to review this interesting approach to the use of risk questionnaires for disease identification by CHA. I hope the comments are useful.

Author’s comments: Thanks a lot and your comments improved a lot our paper.

Thank you for your consideration. I look forward to hearing from you.

Sincerely,
