# Supplementary material for: Active search strategies, clinicoimmunobiological determinants and training for implementation research confirm hidden endemic leprosy in inner São Paulo, Brazil
Source: PLoS Negl Trop Dis. 2021 Jun 14;15(6):e0009495. doi: 10.1371/journal.pntd.0009495 (PMC8224878; doi:10.1371/journal.pntd.0009495)
Supplement: Attachment — Submitted filename: Author Responses May 15, 2021.docx [file pntd.0009495.s003.docx]

May 15, 2021.

Paul J. Converse

Associate Editor

PLOS Neglected Tropical Diseases

Ana LTO Nascimento

Deputy Editor

PLOS Neglected Tropical Diseases

Sub: PNTD-D-21-00357

Dear Editors,

We would like to thank you for all comments and corrections about our manuscript: “Active search strategies, clinicoimmunobiological determinants and training for implementation research confirm hidden endemic leprosy in inner São Paulo, Brazil” because they appointed some important aspects to improve it.

The requested edits were considered and they are in a red in the manuscript.

Thank you for your consideration. I look forward to hearing from you.

Sincerely,
